# Influence of Parylene F Coatings on the Wetting Properties of Soft Polydimethylsiloxane (PDMS)

**DOI:** 10.3390/ma16051938

**Published:** 2023-02-26

**Authors:** Fadoua Mayoussi, Ali Usama, Niloofar Nekoonam, Ivonne Knauer, David Böcherer, Bastian E. Rapp, Dorothea Helmer

**Affiliations:** 1 Laboratory of Process Technology, NeptunLab, Department of Microsystem Engineering (IMTEK) University of Freiburg, 79110 Freiburg im Breisgau, Germany; 2 Institute for Macromolecular Chemistry, University of Freiburg, 79104 Freiburg im Breisgau, Germany; 3 Freiburg Materials Research Center (FMF), University of Freiburg, 79104 Freiburg im Breisgau, Germany; 4 Freiburg Center of Interactive Materials and Bioinspired Technologies (FIT), University of Freiburg, 79110 Freiburg im Breisgau, Germany

**Keywords:** soft wetting, polydimethylsiloxane, adaptive wetting, free oligomers, parylene F

## Abstract

Understanding the wettability of soft surfaces is of key importance for the development of protective and repellent coatings and controlling droplet dynamics when required. There are many factors that affect the wetting and dynamic dewetting behavior of soft surfaces, such as the formation of wetting ridges, the adaptive behavior of the surface caused by the interaction of the fluid with the surface, or the presence of free oligomers that are washed out of the soft surface. In this work, we report the fabrication and characterization of three soft polydimethylsiloxane (PDMS) surfaces with elastic moduli ranging from 7 kPa to 56 kPa. The dynamic dewetting behavior of liquids with different surface tensions was studied on these surfaces, and the data show soft and adaptive wetting behavior of the soft PDMS, as well as the presence of free oligomers. Thin layers of Parylene F (PF) were introduced to the surfaces and their influence on the wetting properties was studied. We show that the thin layers of PF prevent adaptive wetting by preventing the diffusion of liquids into the soft PDMS surfaces and by causing the loss of the soft wetting state. The dewetting properties of the soft PDMS are enhanced, leading to low sliding angles of ≤10° for water, ethylene glycol, and diiodomethane. Therefore, the introduction of a thin PF layer can be used to control wetting states and to increase the dewetting behavior of soft PDMS surfaces.

## 1. Introduction

Soft materials, such as cells, soft tissues, and rubber are omnipresent in nature. Inspired by these materials, engineering synthetic materials showing similar properties has been the objective of many researchers in the last decade. Controlling the wetting on soft materials is of great importance for their use in different fields such as coatings [1] and three-dimensional (3D) printing [2]. Contrary to the wetting concept on rigid surfaces, which was explained by Young’s law based on a force balance of interfacial tensions that determines the equilibrium contact angles at the three-phase contact line (TPCL) [3], wetting on soft surfaces is controlled by other parameters: the elasticity of the material and the liquid’s surface tension. When a liquid droplet is placed on a soft substrate, a microscopic deformation, a so-called wetting ridge, occurs at the TPCL. The geometry of the generated ridge is governed by a balance of surface tensions, interfacial tensions, and substrate elasticity, and it significantly influences the dynamic wetting behavior [4,5,6,7,8]. The movement of a liquid droplet on such soft surfaces undergoes different spreading behaviors, such as viscoelastic braking and stick breaking, which is controlled by the surface softness, spreading rate, wetting ridge height, and layer thickness [6,7,9,10]. Silicone elastomers, such as crosslinked polydimethylsiloxane (PDMS), have been widely used to study the concept of soft wetting. PDMS is known for its high transparency, biocompatibility and flexibility, with a broad range of applications fields from sensors and medical devices to microfluidic devices [11,12,13,14]. Soft PDMS substrates with elastic moduli in the range of kilopascals (kPa) can be easily prepared by adjusting the ratio between crosslinker and monomer. Typically, PDMS elastomers have uncrosslinked molecules inside the crosslinked network, which swell the network and affect the wetting behavior by segregating to the surface [15,16,17]. Wong et al. reported that PDMS surfaces adapt upon wetting by water, which is a result of the free oligomers in the polymer matrix that move up to the solid-liquid interface. In other words, when a droplet is placed onto a PDMS surface, the free oligomers are pulled to the surfaces, causing a reversible transformation of the PDMS from its dry state to a lubricated one [15]. It can be concluded that the wetting and dewetting of soft PDMS samples depend on various factors, such as the history of the sample, the number of free oligomers trapped in the polymer matrix and the time from the droplet deposition to the measurement. Moreover, soft materials are prone to interactions with the wetting phase, which influences their wetting properties. Therefore, control of the wetting states on PDMS is demanding and requires standard procedures for reliable results. Thus, the use of protective films can be a useful tool to control and enhance the wetting properties of soft PDMS. 

Parylene is a popular barrier material that can be easily applied to various substrates via chemical vapor deposition (CVD). Parylene coatings are completely conformal, transparent, and chemically stable. They can be used as protective coatings for various surfaces [18,19,20,21,22,23,24]. Parylene F (PF) has lately emerged as a great barrier material with good ultraviolet (UV) resistance, higher thermal stability and durability [25,26,27,28,29]. PF has been used to coat various materials, such as silicon [30,31], PDMS [32,33], polyurethanes [34], and metals [35,36]. However, the wettability of PF has not been thoroughly studied [28]. Recently, Han et al. reported the wettability and surface free energy (SFE) of PF surfaces, showing a hydrophobic surface with a water contact angle (CA) of 104° and SFE of 27 mNm^−1^ [32]. The reported SFE value is close to the one of conventional parylene C (PC), making PF a potential replacement for PC in practical applications. Tian et al. reported the chemical vapor deposition of PF on PDMS, which showed low SFE, negative surface charge, and antifouling performance against bacteria and algae, promoting the use of such substrates for antifouling applications in marine environments [33]. Yet to date, there are no studies about the dynamic wetting on PF-coated surfaces. The dynamic wetting of liquids on surfaces in general is of great importance, not only in daily life but in various technological applications, such as protective coatings and droplet manipulation and transportation.

Here we present the fabrication and characterization of the wettability of PF-coated PDMS coatings with different softness. The prepared substrates exhibit elastic moduli in the range of 7 kPa to 56 kPa. The wettability of the soft surfaces prior to the deposition of PF was characterized using liquids with different surface tensions, such as water, ethylene glycol and diiodomethane, and the dependence of the wetting and dewetting behavior on the coating layer thickness was also studied. It was observed that the soft PDMS coatings above 50 µm thickness display soft wetting properties. The presence of uncrosslinked siloxanes and the adaptive properties of the surface by liquid imbibition was demonstrated. The influence of the surface modification of the soft PDMS surfaces by CVD with a thin layer (37 ± 10 nm) of Parylene F is studied. The formation of surface wrinkles by the PF deposition is shown, with wrinkles of different sizes depending on the elastic modulus of the soft PDMS surface. The PF also prevents the state of adaptive wetting by preventing the diffusion of wetting liquid into the surface. Depending on the wrinkle size, excellent dewetting properties can be achieved with sliding angles of ≤10° for water, ethylene glycol, and diiodomethane.

## 2. Materials and Methods

### 2.1. Materials

SF00 2k-Silikon was purchased from SILIKONFABRIK (Bad Schwartau, Germany) and Parylene F dimer was purchased from Fluorochem (United Kingdom). (Vinylmethylsiloxane)-dimethylsiloxane copolymer, trimethylsiloxane terminated (XG 0677), Poly (dimethylsiloxane) vinyldimethylsiloxy terminated (DMS V31), (Methylhydrosiloxane) dimethylsiloxane copolymer trimethylsiloxyl terminated (HMS 151), and Platinum-divinyltetramethylsiloxane complex in xylene (SIP 6831.1 4) were purchased from Abcr (Karlsruhe, Germany). Ethylene glycol, diiodomethane, and rhodamine B (RB) were purchased from Sigma Aldrich (Taufkirchen, Germany). THF was purchased from Carl Roth (Karlsruhe, Germany). 

### 2.2. Methods

**Preparation of soft PDMS coatings.** The preparation of two sorts of soft PDMS substrates (Bioclear and commercially available two-components SF) was previously reported [37]. Briefly, soft Bioclear substrates were fabricated by mixing a vinylsiloxane prepolymer (XG 0677, DMS V31) and a hydrosiloxane curing agent (HMS 151) with a platinum catalyst, with the precise amounts being previously reported [37]. Commercial soft PDMS substrates were prepared using crosslinked SF mixed at a weight ratio of 50:1 of pre-polymer to crosslinker, respectively. For comparison, pristine SF substrate was also prepared in its standard ratio (1:1) as a benchmark to better understand the effect of softness on the wetting properties. Thin PDMS films (5–50 μm) were prepared via a spin-coating process and thicker films (250 μm) were prepared using the casting method. The thickness of the thin PDMS coatings was measured using a White Light Interferometer (WLI) of the NewView 9000 (Zygo, Middlefield, CT, USA) type, whilst the thickness of the casted 250 µm coatings was measured using a Heindenhain MT 60 M length gauge. 

**Gel permeation chromatography (GPC).** Prior to the measurements, the PDMS substrates were dissolved/swollen in the eluent, which is THF. The solution (100 µL) was filtered through a 1 µm filter, injected (100 µL injection volume), and chromatographed on four columns: SDV Precolumn, SDV 100 Å, SDV 1000 Å and SDV 10000 Å (PSS Standards, Mainz, Germany). The measurements were performed at a flow rate of 1.0 mL at 22.5 °C and polystyrene standards were used for calibration. 

The data were evaluated using PSS WINGPC UniChrom (PSS Polymer Standards Service GmbH, Mainz, Germany). In the case of a bimodal molecular weight distribution, the integration limit was set as the minimum value in the area between the two peaks. 

**Softness measurements.** The elastic modulus of the PDMS substrates was measured via shear rheology measurements using HAAKE Modular Advanced Rheometer System of type MARS 2 (Thermo Scientific, Germany). The procedure was reported in our previous recent work [37].

**Confocal microscopy.** The deformation of the surfaces upon contact with liquid droplets was visualized using an inverted confocal microscope of type SP8 (Leica, Germany) with a 20X objective with oil immersion. The laser was set at a wavelength of 535 nm and a HyD4 detector was set in the range of 540–650 nm to detect the droplet fluorescence. A 5 μL water drop was dyed with rhodamine B (20 µg/mL) and was placed on the sample. Then, several images were taken on the Z axis.

**Contact angle measurements.** The contact angle (CA) measurements were performed using the optical contact angle measurement system OCA 15 (DataPhysics Instruments, Filderstadt, Germany). For this, 5 µL droplets of water, ethylene glycol, and diiodomethane were measured. For the sliding angle (SA) measurements, 10 µL droplets were used. The substrates were glued onto the stage using adhesive tape. The stage was then tilted at a speed of 1.24°/s until the angle at which the droplet starts moving was reached. The reported values, both CA and SA, are the average of three measurements. 

**Chemical Vapor Deposition of thin Parylene F films.** The chemical vapor deposition (CVD) process of parylene F (PF) thin layers was carried out using PDS 2010 Labcoater (Specialty Coating System (SCS), KISKO, Indianapolis, IN, USA). The parameters were previously reported [37]. The PF dimer was vaporized at 150 °C, then transformed to gaseous monomers in the pyrolysis oven at 650 °C and deposited onto the PDMS surfaces at room temperature. The deposition was carried out with a pressure of 8–16 mbar. To determine the thickness of the deposited PF layers, PF was also deposited on silicon wafers and the thickness was determined using an ellipsometer of type SE 400adv (Sentech Instruments GmbH, Berlin, Germany).

**Environmental scanning electron microscopy (ESEM).** To investigate the wetting state model, ESEM measurements of condensed water droplets on the PF-PDMS surfaces were performed using FEI Quanta 250 FEG. To achieve the condensation process, the temperature was controlled at ~2 °C. At the start, the pressure in the chamber was ~345 Pa. At the selected temperature, water is in its liquid form at atmospheric pressure and gaseous at lower pressures. The water vapor pressure is increased until the liquid-vapor phase-boundary is exceeded resulting in the condensation of water (~750 Pa). Conversely, the sample can be dried again by lowering the pressure. 

## 3. Results

### 3.1. Soft PDMS Coatings and Free Oligomers 

PDMS coatings with different softness were successfully prepared using filler-free, self-mixed Bioclear [13] and commercially available two-component PDMS (SF), which contains silicon dioxide (SiO_2_) as a filler. The softness of SF substrates was achieved by varying the ratio of components A and B from (1:1) to (50:1). The softness of Bioclear was modified by using different amounts of prepolymer and curing agent. The moduli of the resulting three types of PDMS (SF, Bioclear B, and Bioclear C) were determined by shear rheology (see Table 1), showing that low elastic moduli between 7 kPa and 56 kPa were achieved.

**Figure 1:**
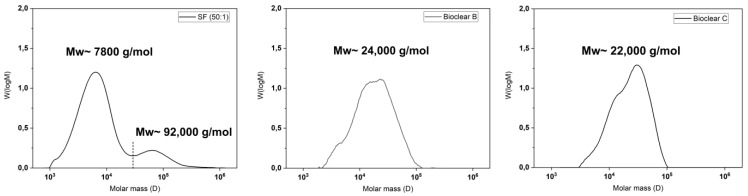
Gel-permeation chromatography (GPC) elution spectrums of normalized distributions vs. molar mass of components of SF, Bioclear B, and Bioclear C showing the average molecular weight of free oligomers. All substrates were cured for 4 h at 65 °C and consecutively placed in THF to elute the unreacted species. For comparison, the individual components of the PDMS types prior to mixing were analyzed (see Figure A1). In the case of SF, the result shows the loss of low-molecular weight species (7800 g/mol) and high-molecular weight species (92,000 g/mol). The dashed line represents the integration limit. In the case of the Bioclear types, the results show medium molecular weight (24,000 g/mol and 22,000 g/mol, respectively).

**Figure A1:**
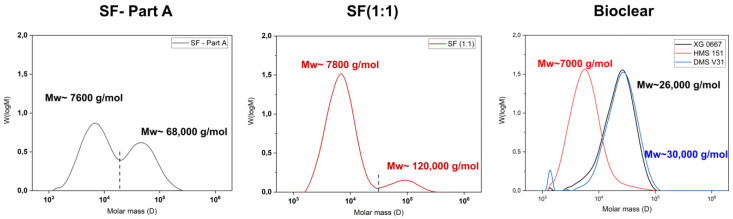
Gel-permeation chromatography (GPC) elution spectrums of normalized distributions vs. molar mass of components of SF (Part A), crosslinked SF (1:1), and the components of Bioclear (XG 0677, HMS151, DMS V31). The dashed line in both SF-Part A and SF (1:1) represents the integration limit.

It is known that the hydrosilylation reaction that takes place upon mixing of PDMS components can be incomplete. Therefore, when fluids are in contact with PDMS, oligomers can be washed out of the material [38]. These free oligomers can alter the results of the wetting analysis of PDMS surfaces [15,17]. Such oligomers are therefore removed prior to wetting experiments by washing. To test for free oligomers in SF, Bioclear B, and Bioclear C, soft PDMS samples were immersed in THF, and the supernatant was analyzed by gel permeation chromatography (GPC). Figure 1 shows the resulting chromatograms of the soft PDMS, indicating a loss of material for all three types of soft PDMS. Commercial SF (50:1) shows a similar molecular distribution as Sylgard 184, another commercially available type of PDMS [15]. SF leaches species of low average molecular weight 7800 g/mol as well as high molecular weight (92,000 g/mol), while the prepared Bioclear PDMS leaches mainly species of medium average molecular weight (24,000 g/mol and 22,000 g/mol for Bioclear B and C respectively). For comparison, the molecular weight of free oligomers in regularly prepared hard SF (ratio A:B = 1:1) and the molecular weight of the prepolymers of Bioclear was also investigated (see Figure A1). For commercial SF, the low-molecular weight peak at 7600 g/mol corresponds well with the molecular weight of the monomer, indicating completely unreacted part A could be present in the cured soft PDMS prior to washing. For Bioclear, the average molecular weight of the free oligomers does not match the average molecular weight of the PDMS components, indicating that Bioclear PDMS components react to form longer chains that are not connected in the PDMS network.

### 3.2. Wetting and Dewetting Behavior of Soft PDMS Substrates

**Figure 2:**
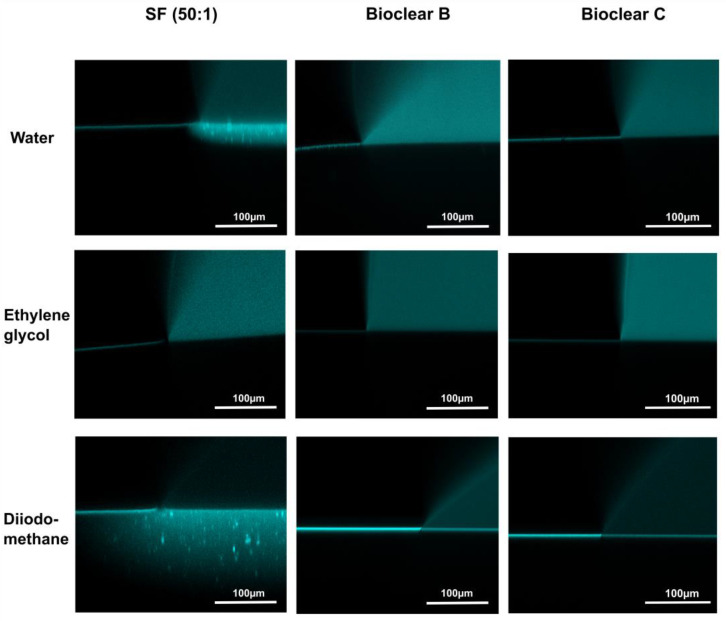
Confocal microscopy images of the three-phase contact line area of 5 µL droplets of water, ethylene glycol, and diiodomethane on soft PDMS types SF and Bioclear B and C. Droplets were dyed with rhodamine B to increase visibility. Water and diiodomethane diffuse into the soft SF surface as indicated by the bright halo underneath the droplet. On Bioclear, no diffusion of fluids is observed.

**Figure A2:**
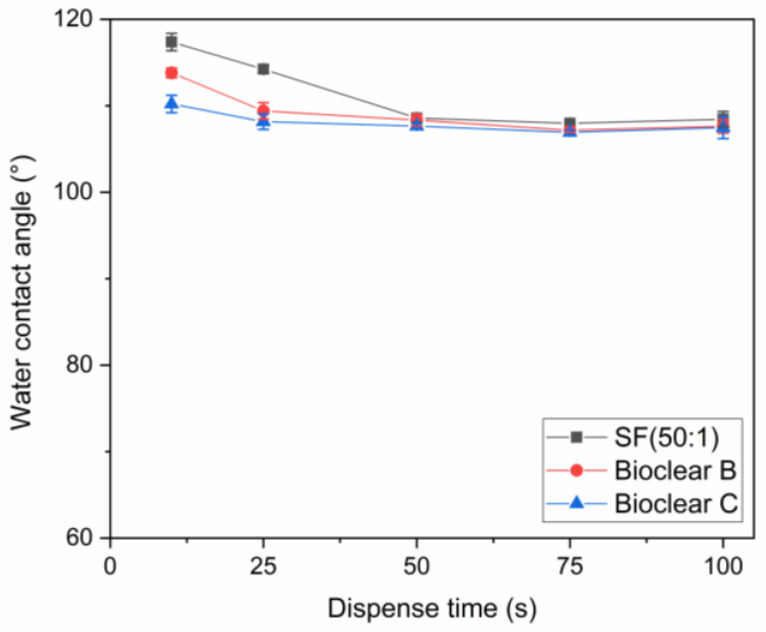
Measured CA of water in dependence on the dispense time.

**Figure A3:**
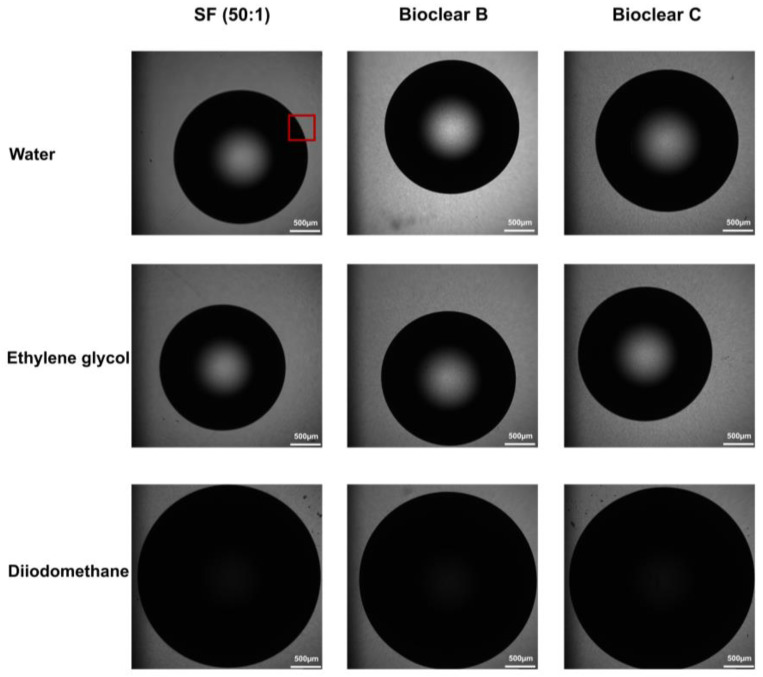
Brightfield microscopy images of 5 µL droplets of water, ethylene glycol and diiodomethane on the soft PDMS surfaces. The red rectangle highlights the three-phase contact line area where the confocal microscopy images were taken in the Z-axis.

To test the wetting behavior of the prepared soft PDMS coatings, CA measurements were performed using three different liquids: water, ethylene glycol (EG), and diiodomethane (DI). The three liquids were chosen as they differ in their surface tensions as well as their polar and dispersive components according to Owens, Wendt, Rabel and Kaelble (OWRK) (See Table A1) [39]. The wetting characterizations were performed on the soft substrates containing the free uncrosslinked siloxanes. Prior to the measurements, the time required for a droplet to reach its equilibrium on the soft surfaces was tested. 5 µL water droplets were observed over 100 s after being placed on the surfaces (see Figure A2). The static CA values stayed constant after 45 s from dispensing, thus all measurements were performed after 45 s. Static CAs were measured on soft PDMS substrates with different layer thicknesses between 5 µm and 250 µm (see Table A2). For the three soft PDMS types, the static CA of water, EG, and DI did not change with varying thicknesses. Thickness-dependent wetting behavior is characteristic of soft materials [9] as the elastic modulus increases with decreasing layer thickness [40,41], and the influence of the wetting ridges on the droplet movement becomes relevant. To determine whether the PDMS surfaces display this characteristic, the dependence of the layer thickness on the dynamic wetting and dewetting behavior was tested for soft PDMS coatings of different thicknesses. The sliding angles (SA) of water and EG on the soft PDMS showed dependence on the layer thickness (see Table A3): above a material thickness of 50 µm, a significant increase in SA is observed for water and EG. The SA of DI remained unaffected by the thickness. This is likely due to the diffusion of DI into the surface, causing a brush-like wetting state in the very sporadically crosslinked PDMS gel. PDMS-brushes are notorious for their good performance in dynamic wetting [42,43,44]. This adaptive wetting behavior of soft PDMS substrates is well-known and refers to the effect of the wetting fluid altering the surface of the PDMS by diffusion of the fluid into the material [15,17]. Microscopically, the effect of solvent uptake can be visualized using confocal microscopy with dyed fluids, by observing the PDMS surface underneath the deposited fluid droplet at the three-phase contact line (contact line between gas phase, droplet, and surface). To test this, water, EG, and DI were dyed with rhodamine B, 5 µL droplets were applied to the soft PDMS surface, and confocal microscopy images were taken (see Figure 2). It is observed that RB diffuses into the surface of the soft PDMS SF, but not into Bioclear B and C coating. This could be due to the very low modulus of SF, making it prone to diffusion effects, or due to the SiO_2_ fillers contained in this commercial PDMS type. As a reference, brightfield images of the different liquid droplets on the soft surfaces were taken to highlight the three-phase contact line shown in the confocal microscopy images (See Figure A3).

**Figure 3:**
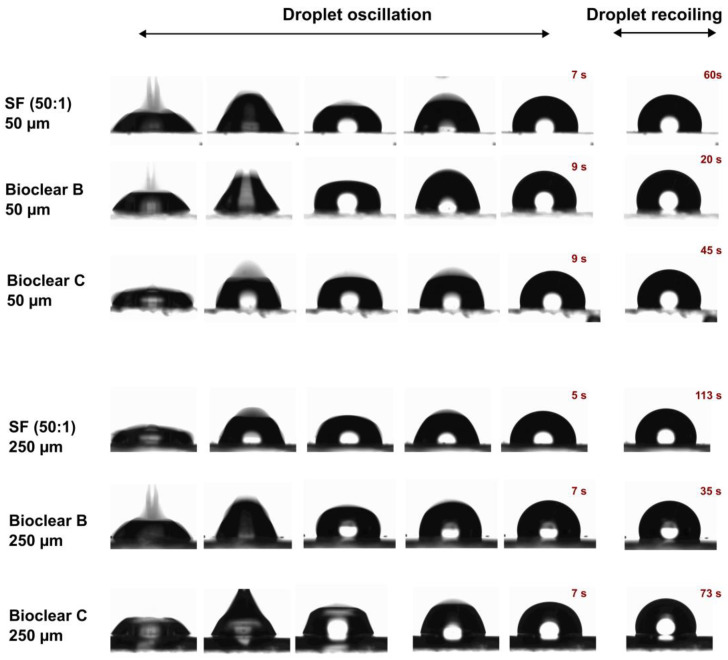
Image sequences of water droplet impacting on the soft PDMS surfaces: SF (50:1), Bioclear B, and Bioclear C with different thicknesses (50 µm and 250 µm).

In addition to the wettability, the effect of the surface softness on drop dynamics is usually investigated. For this, the impact of water droplets on the soft PDMS surfaces at a velocity of 0.25 cm/s was tested. Figure 3 shows image sequences of a water droplet impacting on SF (50:1), Bioclear B and Bioclear C surfaces with two different thicknesses 50 µm and 250 µm. As above-mentioned, it was concluded that the soft PDMS show a thickness-dependent wettability, i.e., the elastic modulus decreases by increasing the thickness. In brief, the soft PDMS coatings with a thickness of 250 µm show soft wetting behavior, whereas the 50 µm coatings show a substrate-dependent wetting behavior. It was previously reported that the droplets impacting on soft viscoelastic surfaces show two distinct behaviors: oscillation and recoiling/rebounding. These behaviors are significantly influenced by the softness degree [45,46]. Upon contact with the soft PDMS surfaces, a water droplet goes through oscillation at the contact line for few seconds and then starts recoiling until it reaches its equilibrium state. A difference in the oscillation and recoiling phases was observed. Surfaces showing soft wetting behavior exhibited reduced oscillation time and longer recoiling time in comparison to the surface showing substrate-dependent wetting behavior. This difference was also observed when comparing Bioclear B, which shows the highest elastic modulus amongst the samples (E~56 kPa) to Bioclear C (E~15 kPa) and SF (50:1) (E~7 kPa). Bioclear B, in both thicknesses, showed the shortest droplet recoiling time, whereas SF (50:1) showed the longest recoiling time. This behavior might be a result of the formed wetting ridge at the contact line inhibiting the fast recoiling of droplets on the softer surfaces. 

### 3.3. Wetting Behavior of Parylene F Coated Soft PDMS

**Figure 4:**
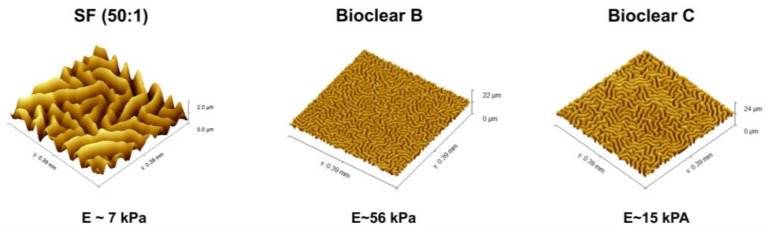
Visualization of the wrinkles. WLI image of the generated wrinkles on SF, Bioclear B, and Bioclear C and their corresponding profile. Images show different z-scales: the height of the wrinkles on SF and Bioclear is similar, but they vary in their lateral dimensions with one wrinkle on SF at approximately 45 µm and 20 µm on Bioclear.

Parylene is a well-known sealant for surfaces, being widely used in the industry for coatings of PCB boards [47,48]. Thin layers of Parylene are effective liquid and gas barriers [21,24,29]. Pristine smooth layers of PF show static CA of 92°, 66°, and 50° and SA of 36°, 31°, and 9° for water, EG, and DI, respectively (see Table A4). Here, the influence of a thin parylene coating on the wetting properties of soft PDMS surfaces of 50 µm and 250 µm thickness was tested to reduce the effects of free oligomers and thus to eliminate the effects of adaptive wetting. A thin PF layer with a thickness of 37 ± 10 nm was deposited onto the surfaces by CVD. The deposition process resulted in a change in the surface topography generating micro-wrinkles with different dimensions (see Figure 4). The wrinkle profiles and surface roughness of the prepared surfaces were previously reported [37]. Wrinkles show almost a similar depth for all samples which is in the range of ~2 µm, but wrinkles on SF have larger lateral dimensions than on Bioclear B and C. Wrinkles on SF are in the range of ~45 µm, whereas wrinkles on Bioclear are in the range of ~20 µm (See Table A5). 

**Figure 5:**
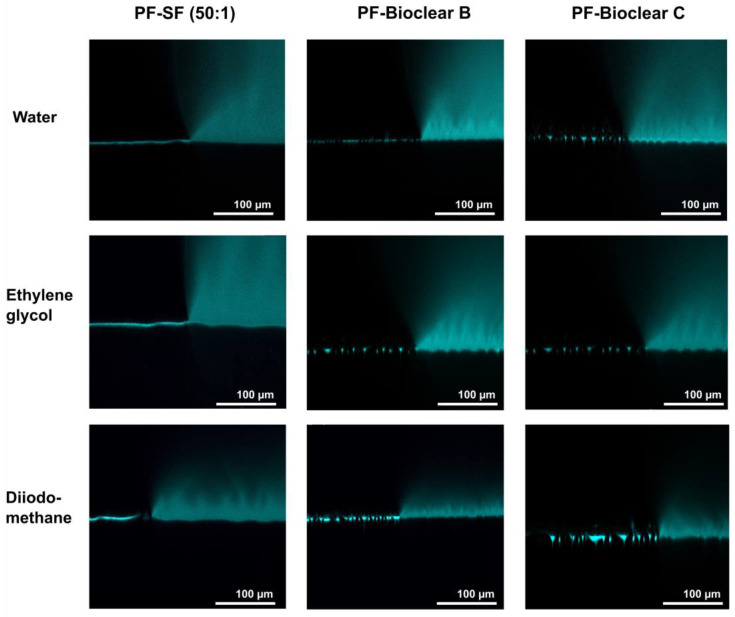
Confocal microscopy images of the three-phase contact line area of 5 µL droplets of water, ethylene glycol, and diiodomethane on soft PDMS types SF and Bioclear B and C coated with a thin layer of Parylene F. Droplets were dyed with rhodamine B to increase visibility. The PF coating prevents the diffusion of fluid into the soft material (compare Figure 2). The wrinkles formed by the PF coating are clearly visible on the surfaces.

**Figure A4:**
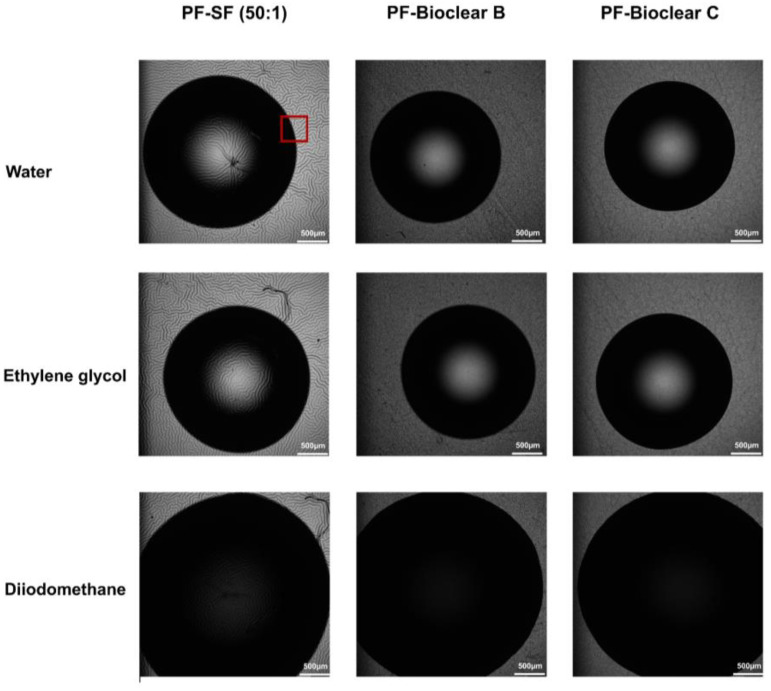
Brightfield microscopy images of 5µL droplets of water, ethylene glycol and diiodomethane on the PF-PDMS surfaces. The red rectangle highlights the three-phase contact line area where the confocal microscopy images were taken in the Z-axis.

First, to test the ability of PF-coatings to prevent the diffusion of fluids into the soft surface, confocal microscopy images of fluid droplets dyed with Rhodamine B were taken on the PF-coated soft PDMS (see Figure 5). The images show the wrinkled structure of the surface and confirm that no diffusion of liquid takes place, as indicated by the absence of a halo underneath the droplet. Figure A4 shows brightfield images of water, EG, and DI droplets on the PF-coated surfaces highlighting the three-phase contact line shown in the confocal microscopy images.

**Figure 6:**
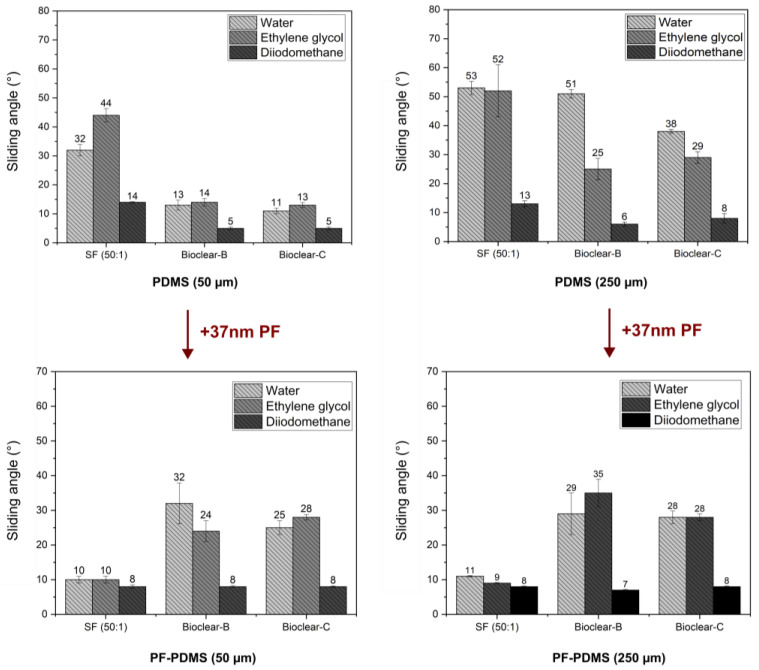
Dewetting behavior of water, ethylene glycol and diiodomethane on soft and PF-coated PDMS with a thickness of 50 µm and 250 µm. The wetting behavior is independent of the PDMS layer thickness, indicating that the PF-coating eliminates the effect of soft wetting. The SA values of PF-Bioclear correspond well with the SA values of pristine parylene F on a smooth surface, indicating that surface chemistry is the main influence on the wetting behavior. For SF, SA values are significantly decreased on PF-coated PDMS, indicating that the wrinkled surface structure is the main influence on the wetting behavior of PF-SF.

**Figure A5:**
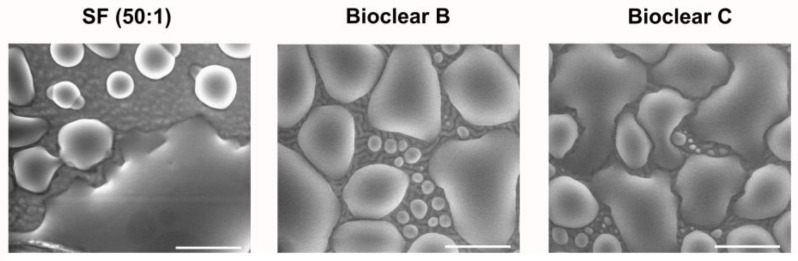
ESEM images of condensed and coalesced water droplets on PF-PDMS surfaces during the drop growth phase. The Droplets adhere to the surfaces and spread cross the wrinkles while exhibiting low contact angles. (Scale bar: 100 µm).

The wetting properties of wrinkled PF-coated soft PDMS surfaces are potentially governed by three effects: the softness of the surface, surface structuring, and surface chemistry. To evaluate the influence of the individual effects, the wetting properties of the PF-coated soft PDMS were investigated. For the investigation of the droplet shape on the PF-PDMS surfaces, ESEM measurements were conducted (See Figure A5). Furthermore, CA and SA measurements were performed using water, EG, and DI on 50 µm and 250 µm thick layers of soft PDMS. When comparing the results of PF-coated soft PDMS to uncoated soft PDMS, it was found that the static CA values of water did not change (see Table A6). For EG, the static CA was slightly decreased on SF (from an average of 92° on pristine soft SF to 84° on PF-coated SF). For DI a decrease in static CA was observed for all PDMS types with a static CA of ~75° on all soft PDMS surfaces to ~60° on the PF-PDMS surfaces. In terms of SA, it was observed that there was no significant change when the thickness of the PF-coated PDMS layer was increased (see Figure 6 and values are listed in Table A7). This indicates that a thin PF coating eliminates the thickness-dependent effect of soft wetting on all surfaces. When comparing the SA of pristine soft PDMS to PF-coated PDMS, an increase was observed for Bioclear B and C. The increased values of PF-coated Bioclear correspond well with the SA values of smooth PF coatings on glass (see Table A4). This indicates that the surface chemistry is most relevant for the wetting behavior of PF-coated Bioclear. For the PF-SF surface, the deposition of the PF layer led to significantly decreased SA, with values even below the SA values of smooth PF coatings. This indicates that for PF-coated SF the surface structure plays a more distinct role in the wetting properties, indicating that the lateral size of the wrinkles is also crucial for PF-coated soft PDMS. Thus, thin PF-coatings can be used to enhance the dewetting properties of soft PDMS surfaces.

## 4. Conclusions

PDMS can be used to reach different wetting states, such as soft and adaptive wetting, depending on its moduli and chemical structure. In this paper, the influence of a thin layer of Parylene F was used to modify and analyse the corresponding wetting states. Three types of soft PDMS substrates (SF, Bioclear B, and Bioclear C) of different elastic moduli were prepared. The soft wetting regime of fluids on the soft PDMS surfaces was confirmed by the thickness-dependent sliding behaviour of the wetting fluids. The soft PDMS types were tested for their content of free oligomers, which influence the wetting behaviour. Free oligomers were found in all PDMS types. Additionally, the diffusion of wetting liquids into the PDMS surface was shown by confocal microscopy, indicating a state of adaptive wetting for water and diiodomethane on soft PDMS SF. Thin layers of Parylene F (PF) were deposited onto the soft PDMS and the wetting behaviour was analysed. The deposition of PF induced the formation of surface wrinkles of different dimensions depending on the elastic modulus of the soft PDMS. The PF coating successfully prevented the diffusion of fluid into the PDMS surface as shown by confocal microscopy. However, PF-coated soft PDMS no longer displays thickness-dependent dynamic wetting, indicating that the soft wetting state is lost by the PF coating. Depending on the size of the surface wrinkles induced, the droplet sliding angles of soft PDMS can be increased or decreased. For smaller lateral wrinkle dimensions, the wetting properties of PF-coated PDMS are similar to the properties of pristine, smooth PF. For larger wrinkle dimensions, the dewetting properties of the soft PDMS surface are significantly enhanced compared to the soft PDMS surface and to pristine PF. Thus, the deposition of PF can significantly enhance the wetting properties of soft PDMS, reaching low sliding angles of ≤10° for water, ethylene glycol, and diiodomethane.

## Figures and Tables

**Table 1:** Measured elastic moduli of the soft PDMS substrates.

PDMS Substrates	SF (50:1)	Bioclear-B	Bioclear-C
**E-modulus (kPa)**	7 ± 0.2	56 ± 6	15 ± 1

## Data Availability

The data is contained within the article.

## Appendix A

**Table A1:** Polar and dispersive components of the surface tension of water, ethylene glycol, and diiodomethane according to the WORK method [39].

	Water	Ethylene Glycol	Diiodomethane
**Total** (mNm^−1^)	72.8	48	50.8
**Polar** (mNm^−1^)	51	19	0
**Dispersive** (mNm^−1^)	21.8	29	50.8

**Table A2:** CA values of water, ethylene glycol, and diiodomethane on soft PDMS surfaces with different thicknesses. The CA values did not show significant differences with varying thickness.

Thickness (µm)	SF			Bioclear B			Bioclear C		
	Water (°)	E.G (°)	D.I (°)	Water (°)	E.G (°)	D.I (°)	Water (°)	E.G (°)	D.I (°)
**5**	109 ± 2	94 ± 2	72 ± 2	105 ± 1	85 ± 1	71 ± 1	107 ± 1	84 ± 1	72 ± 1
**15**	110 ± 1	91 ± 2	71 ± 1	107 ± 1	85 ± 2	72 ± 2	105 ± 1	85 ± 1	70 ± 1
**25**	110 ± 1	90 ± 3	73 ± 1	106 ± 1	84 ± 1	70 ± 1	105 ± 1	85 ± 1	71 ± 1
**50**	111 ± 1	92 ± 3	74 ± 2	105 ± 1	86 ± 1	73 ± 1	105 ± 1	83 ± 1	71 ± 2
**250**	109 ± 1	94 ± 2	72 ± 2	106 ± 1	84 ± 2	70 ± 1	105 ± 1	84 ± 1	72 ± 2

**Table A3:** SA values of water, ethylene glycol, and diiodomethane of soft PDMS surfaces with different thicknesses. Above 50 µm a significant increase in SA is observed for water and EG on all PDMS types. This indicates that the substrate softness plays a key role and confirms the soft wetting state on the substrates.

Thickness (µm)	SF (50:1)			Bioclear B			Bioclear C		
	Water (°)	E.G (°)	D.I (°)	Water (°)	E.G (°)	D.I (°)	Water (°)	E.G (°)	D.I (°)
**5**	31 ± 3	34 ± 3	20 ± 3	10 ± 2	10 ± 1	4 ± 2	11 ± 2	12 ± 3	4 ± 1
**15**	27 ± 4	33 ± 5	15 ± 3	11 ± 2	12 ± 1	6 ± 1	12 ± 2	11 ± 1	5 ± 1
**25**	33 ± 4	30 ± 4	12 ± 2	11 ± 2	12 ± 2	5 ± 1	11 ± 3	12 ± 2	4 ± 1
**50**	33 ± 3	34 ± 3	13 ± 3	12 ± 2	14 ± 2	5 ± 1	10 ± 1	11 ± 2	5 ± 1
**250**	54 ± 3	52 ± 8	14 ± 3	51 ± 5	25 ± 4	5 ± 1	35 ± 3	28 ± 3	8 ± 2

**Table A4:** CA and SA of water, ethylene glycol, and diiodomethane on a PF surface.

	PF Coating on a Glass Slide		
	Water	Ethylene Glycol	Diiodomethane
**CA (°)**	92 ± 3	66 ± 1	50 ± 1
**SA (°)**	36 ± 3	31 ± 3	9 ± 2

**Table A5:** Dimensions of the wrinkles generated on the soft PDMS substrates (SF, Bioclear B, and Bioclear C) upon deposition of a thin layer of PF.

Layer Thickness (µm)	Wrinkle Dimension Depth (µm)			Wrinkle Dimension Lateral (µm)		
	SF (50:1)	Bioclear B	Bioclear C	SF (50:1)	Bioclear B	Bioclear C
**50**	1 ± 0	2 ± 1	2 ± 0	42 ± 8	14 ± 3	14 ± 6
**250**	2 ± 1	3 ± 1	3 ± 1	40 ± 8	13 ± 7	19 ± 8

**Table A6:** CA values of water, ethylene glycol, and diiodomethane on PF-coated soft PDMS surfaces with different thicknesses. The CA values did not show significant differences with varying thicknesses. Compared to pristine soft PDMS, the values of DI decreased from ~72° to ~60° on both Bioclear surfaces.

Thickness (µm)	SF			Bioclear B			Bioclear C		
	Water (°)	E.G (°)	D.I (°)	Water (°)	E.G (°)	D.I (°)	Water (°)	E.G (°)	D.I (°)
**50**	109 ± 1	84 ± 1	67 ± 1	110 ± 2	83 ± 1	61 ± 1	108 ± 2	82 ± 1	61 ± 0
**250**	108 ± 1	84 ± 1	68 ± 2	108 ± 1	83 ± 1	59 ± 0	109 ± 1	83 ± 0	60 ± 1

**Table A7:** SA values of water, ethylene glycol, and diiodomethane on PF-coated soft PDMS surfaces with different thicknesses.

Thickness (µm)	SF (50:1)			Bioclear B			Bioclear C		
	Water (°)	E.G (°)	D.I (°)	Water (°)	E.G (°)	D.I (°)	Water (°)	E.G (°)	D.I (°)
**50**	10 ± 1	10 ± 1	8 ± 1	32 ± 6	24 ± 3	8 ± 0	25 ± 2	28 ± 1	8 ± 0
**250**	11 ± 1	9 ± 1	8 ± 1	29 ± 6	35 ± 4	7 ± 0	28 ± 2	28 ± 1	8 ± 0
